# Advancing Neurological Rehabilitation: The BRAIN Framework for Clinical Reasoning in Neurophysiotherapy

**DOI:** 10.3390/brainsci16020235

**Published:** 2026-02-18

**Authors:** Alfredo Lerín-Calvo, Raúl Ferrer-Peña, Sergio Lerma-Lara

**Affiliations:** 1 Grupo de Investigación de Neurociencias Aplicadas a la Rehabilitación (GINARE), 28923 Alcorcón, Spain; alfredo.lerin@lasallecampus.es; 2 Department of Physiotherapy, Centro Superior de Estudios Universitarios La Salle, Universidad Autónoma de Madrid, Aravaca, 28023 Madrid, Spain; sergio.lerma@lasallecampus.es; 3 Clinico-Educational Research Group on Rehabilitation Sciences (INDOCLIN), Centro Superior de Estudios Universitarios La Salle, Universidad Autónoma de Madrid, 28023 Madrid, Spain; 4 Centro de Salud Entrevías, Gerencia Asistencial de Atención Primaria, Servicio Madrileño de Salud, 28053 Madrid, Spain; 5 University Clinic, Centro Superior de Estudios Universitarios La Salle, Universidad Autónoma de Madrid, Aravaca, 28023 Madrid, Spain

**Keywords:** clinical reasoning, neurological rehabilitation, physiotherapy, ICF, biopsychosocial model, motor control, BRAIN framework

## Abstract

**Highlights:**

**What are the main findings?**

- The BRAIN (framework is proposed as a clinical reasoning model for neurophysiotherapy that integrates the ICF, the APTA patient-management process, and adapted physical-function domains.
- The framework translates biopsychosocial, goal-oriented reasoning into a structured workflow by linking key impairment domains (e.g., motor control, sensory functions, hyperresistance) with standardized assessment options across activity and participation.

**What are the implications of the main findings?**

- The model provides a consistent, shared language that can strengthen interdisciplinary communication, documentation, and clinical decision-making in complex neurological rehabilitation.
- BRAIN offers a teachable template to support clinician training and future validation studies (e.g., expert consensus and real-world implementation pilots), with the potential to optimize functional, patient-centered outcomes.

**Abstract:**

**Background/Objectives**: Clinical reasoning is essential in neurological rehabilitation, in which patient management is complex and multifactorial. However, existing models lack operationalization for neurophysiotherapy practice. This paper proposes the Biopsychosocial Reasoning Approach In Neurophysiotherapy (BRAIN) framework, a unified clinical reasoning model that integrates the International Classification of Functioning, Disability, and Health (ICF) with neurophysiotherapy-specific domains of physical function. **Methods**: The BRAIN framework was developed by integrating previously validated models: the ICF, the American Physical Therapy Association (APTA) patient-management process, and Kisner and Colby’s interrelated aspects of physical function, adapted for neurological populations. The model employs a biopsychosocial, patient-centered, and goal-oriented approach, thereby providing a structured workflow for examination, evaluation, diagnosis, prognosis, intervention, and outcomes assessment. **Results**: The BRAIN framework provides an operationalized mapping between ICF body functions and neurophysiotherapy-specific impairment domains, a clear separation between body functions and body structures, tiered assessment pathways addressing patient heterogeneity, and a unified language for interdisciplinary communication. The model incorporates shared decision-making and goal-oriented reasoning within the clinical workflow. **Conclusions**: The BRAIN framework offers a structured, teachable template for clinical reasoning in neurological physiotherapy. As a theoretical proposal, there is a need for empirical validation through expert consensus and clinical implementation studies.

## 1. Introduction

Clinical reasoning is often regarded as the foundation of clinical practice [1]. As posited by several authors, the construct is best characterized as multidimensional, a perspective that underscores its inherent complexity and integration of disparate domains [2]. It is regarded as a fundamental skillset for healthcare professionals and has been the focus of extensive research by various healthcare professionals [2,3]. Its definition is subject to variation based on the discipline: it is more focused on diagnosis when adopted by physicians or on the decision-making process when used by psychologists, physiotherapists or occupational therapists [4]. However, clinical reasoning can be defined as “the mental process that happens when a healthcare professional encounters a patient and is expected to draw a conclusion about (a) the nature and possible causes of complaints or abnormal conditions of the patient, (b) a likely diagnosis, and (c) patient management actions to be taken” [5].

The clinical reasoning process comprises a multitude of interacting factors, including the patient, the clinician, and the clinical environment. The prevailing categorization of this phenomenon encompasses two primary types: inductive (or forward) reasoning and deductive (or backwards) reasoning [6,7]. Inductive reasoning comprises two subsystems: the first relies on pattern recognition, predominantly driven by the clinician’s expertise and experiential knowledge; the second involves a slower, more effortful analytical thinking process [8]. These two systems are situated on a cognitive continuum, ranging from immediate, intuitive recognition to deliberate and prolonged analytical reasoning [8,9]. Conversely, deductive clinical reasoning involves constructing a mental model, or a set of models, derived from general knowledge, established theoretical principles, and clinical guidelines. The use of these models is predicated on their capacity to facilitate the formulation of hypotheses concerning the patient’s presentation and possible outcomes. Clinicians then systematically evaluate these hypotheses by collecting and analyzing evidence to confirm or refute them. In the absence of disconfirming evidence during clinical assessment, initial mental models are reinforced, thereby supporting valid clinical conclusions and facilitating sound clinical decision-making [10].

Clinical reasoning is an essential competency for physiotherapists. It must be an ongoing process that evolves along the continuum of the patient’s rehabilitation. Furthermore, it must be carried out in a multidisciplinary manner, integrating cognitive, psychomotor and affective skills [4,11]. In the domain of neurologic physiotherapy, we can observe an evolution in the decision-making process. The initial theories, grounded in neurophysiological perspectives, aimed to achieve better rehabilitation outcomes based on personal experiences [12], and subsequently, a more scientific approach emerged within the biopsychosocial model, encompassing a more rigorous and systematic framework [13,14].

However, these most recent clinical reasoning models have not yet been fully explained or applied in clinical settings. They are not adapted to physiotherapy and most are derived from Neurodevelopmental Therapies (NDT) [15], which have yet to demonstrate their efficacy in improving functional outcomes in people with neurological deficits (these limitations are discussed in Section 2) [16,17]. Furthermore, empirical evidence suggests that practicing clinicians, including physiotherapists, often rely heavily on implicit, experience-based heuristics rather than explicit theoretical frameworks [18]. Clinical decision-making in real-world settings is often influenced by personal experience accumulated over years of practice, guidance from mentors and supervisors, and local workplace culture or protocols [18]. While such experiential reasoning can be efficient and contextually appropriate, its reliance on tacit knowledge poses challenges for standardization, education, interdisciplinary communication, and quality assurance [19]. In the domain of neurological rehabilitation, challenges such as patient heterogeneity, complexity, and the need for a multidisciplinary collaboration underscore the importance of a shared, explicit reasoning language [20]. Thus, there is an urgent need for structured frameworks that can link theoretical models with clinical practice—frameworks that are both evidence-based and operationally feasible for real-world implementation [21].

The primary objective of this study was to present an updated and operational model of clinical reasoning specific to neurological physiotherapy, which facilitates decision-making, improves interdisciplinary communication, and optimizes patients’ functional outcomes.

## 2. Foundations and Development of Clinical Reasoning Models in Physiotherapy

In 1970, Brunnstrom published her seminal work “Movement Therapy in Hemiplegia: A Neurophysiological Approach”, basing her hypotheses on experiments carried out on animals at the beginning of the 20th century [22]. These studies provide the fundamental bases for recovery after brain damage. In this context, authors such as Sherrington began to observe the phenotypes of spasticity based on the location and severity of the lesion [23,24,25].

Brunnstrom’s method is predicated on post-stroke synergies that represent primitive reflexes liberated from higher control, thereby suggesting developmental regression [22,26]. She emphasizes the pivotal role of spasticity in the context of paresis, asserting that its elimination should be prioritized as a fundamental step in addressing the condition—a contested position [22]. Research shows limited and debated evidence for physiotherapy’s impact on spasticity [27,28,29].

NDT emerged from this perspective, notably the Bobath concept, defined as a problem-solving framework for assessing and treating function, movement, and tone impairments from central nervous system lesions using key handling points and reflex patterns [30,31]. This approach is characterized as complex, multidimensional, and individualized, emphasizing “how” tasks are performed while avoiding compensatory movements [32].

The clinical reasoning behind the Bobath concept is based on neurophysiological grounds that aim to achieve ‘normal movement’ in individuals with brain damage through the facilitation of movement to enhance motor learning [33]. Therefore, motor control is the primary axis of the reasoning process, which is conceptualized as the continuous interaction between the individual, the task, and the environment. This interaction results in movement through the motor, cognitive, and sensory systems [34,35]. The absence of a clearly defined framework has resulted in significant challenges in defining and researching this phenomenon [15,33]. Moreover, recent evidence does not support its implementation in clinical settings due to its ineffectiveness and because its theoretical foundations contradict the latest trends in motor learning and recovery for patients with neurological deficits [36]. Current approaches emphasize increasing the dose and intensity of treatments while allowing patients to experience some errors, thereby facilitating the acquisition of competencies needed to achieve greater functionality and independence [36,37,38,39].

**Figure 1:**
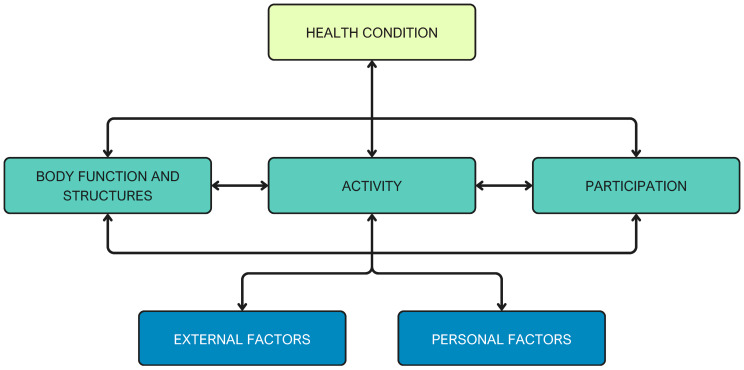
International Classification of Functioning. This model illustrates the reciprocal interactions among health conditions, body functions/structures, activities, participation, and contextual factors (environmental and personal). Each component can influence and be influenced by the others, emphasizing the holistic, biopsychosocial nature of functioning and disability.

In 2001, the International Classification of Functioning, Disability and Health (ICF) was approved by the World Health Organization (WHO), following a global collaboration that provided a comprehensive conceptualization of disability [40,41]. ICF signified a step forward in decision-making based on the biopsychosocial model in physiotherapy [42,43]. The ICF model classifies disability based on four main components. First, the health status of the patient is determined, which refers to the diseases or disorders that affect the patient. Secondly, the concept of “body functions and structures” is introduced. The term “body functions” is defined as the physiological processes of body systems, which also encompasses psychological functions. In contrast, “body structures” refer to the anatomical components of the body, including organs and limbs, along with their respective components. Third, activity is defined as the ability to complete an action or task. Fourth, participation, which involves the patient’s engagement in social situations [26,41] (Figure 1), emphasizes activity and participation as the core concepts of rehabilitation [44].

This tool has promoted the use of a common language among professionals in the domain of neurological rehabilitation. In this domain, collaborative endeavors across disciplines (multidisciplinary, interdisciplinary, or transdisciplinary) are particularly important given the complexity of the patient’s cases [13,36]. Consequently, ICF was introduced as a pivotal element of clinical reasoning in neurological rehabilitation, thereby establishing the patient-centred approach [45].

To this end, goal-oriented approaches have been developed to ensure a patient-centered therapeutic experience. Goal planning is defined as a collaborative process whereby healthcare professionals and service users establish and commit to achieving specific behavioral objectives within a predetermined timeframe [46]. This methodology is operationalized through SMART objectives, which stipulate that therapeutic goals must be Specific, Measurable, Achievable, Realistic, and Time-bound [47].

The development of the Goal Attainment Scale (GAS) was motivated by the necessity to quantify these objectives and to facilitate the translation of this methodology into research settings. [48]. GAS employs a 5-point Likert scale to evaluate the extent of goal attainment. A score of 0 indicates the expected outcome following intervention, scores of +1 and +2 denote outcomes that are greater and much greater than expected, respectively, whereas scores of −1 and −2 indicate outcomes that are less and much less than expected [49].

**Figure 2:**
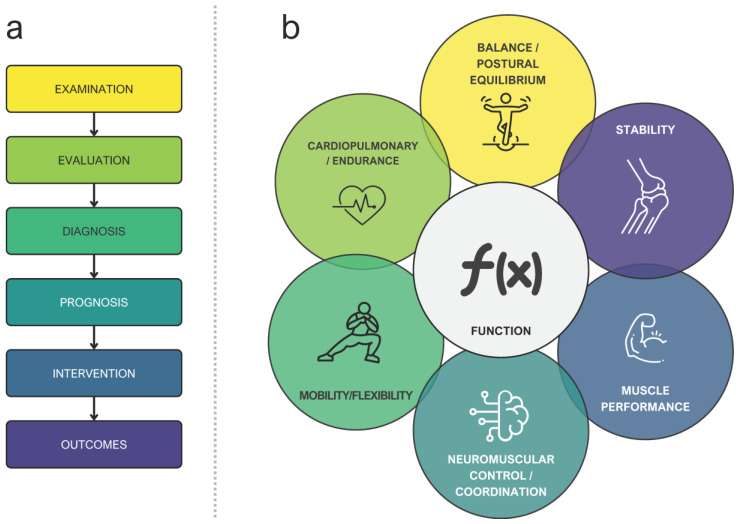
(**a**) Patient management stages by the American Physical Therapy Association. (**b**) Interrelated aspects of function.

Furthermore, in 1995, the American Physical Therapy Association (APTA) developed a physiotherapy practice guideline [50], which established the principles that must be integrated into a model of clinical reasoning specifically oriented to physiotherapy and physiotherapists [51]. The classical APTA clinical reasoning proposal encompasses patient management based on the following stages: examination, evaluation, diagnosis, prognosis, intervention, and outcomes (Figure 2a) [52]. The model under consideration encompasses an evaluation of various domains associated with the patient’s disability, thereby addressing the multidimensional aspects of physical function mentioned by Kisner and Colby [53]: neuromuscular control/coordination; muscle performance; cardiopulmonary/endurance; mobility/flexibility; balance/postural equilibrium; stability, (Figure 2b) [52].

## 3. Current Framework for Clinical Reasoning in Physiotherapy

The *Biopsychosocial Reasoning Approach In Neurophysiotherapy* (BRAIN) framework presented in this section constitutes a theoretical and conceptual proposal developed by integrating models from physiotherapy and rehabilitation that have been previously validated and widely accepted. Specifically, the BRAIN framework aims to unify the classical model of clinical reasoning in physiotherapy promoted by the APTA [52] and WHO decision-making models driven by the ICF [41], adapting them to neurological physiotherapy by incorporating the interrelated aspects of function mentioned by Kisner and Colby [53]. The development of a useful clinical reasoning model applicable to this branch of physiotherapy is predicated on the incorporation of the function model within the ICF. This incorporation would ensure the inclusion of physiotherapy as one of the health professions involved in the multidisciplinary rehabilitation approach for neurological patients. 

### 3.1. Body Functions: Impairment-Level Domains

**Figure 3:**
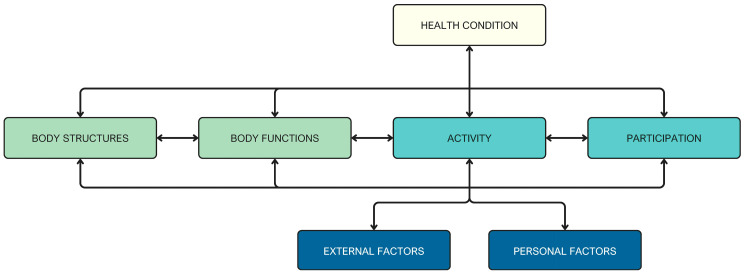
Modified ICF scheme.

According to this paradigm, the interrelated aspects of function (Figure 2b) should be assessed during the examination phase of the classical model and must be included in the ‘body function’ section of the ICF, which refers to deficits in the physiological functions of body systems. This modification entails a refinement of the ICF classical scheme, a process that involves the separation of sections as delineated within the scheme itself. The subsequent adaptation of this scheme to our proposal is illustrated in Figure 3.

**Figure 4:**
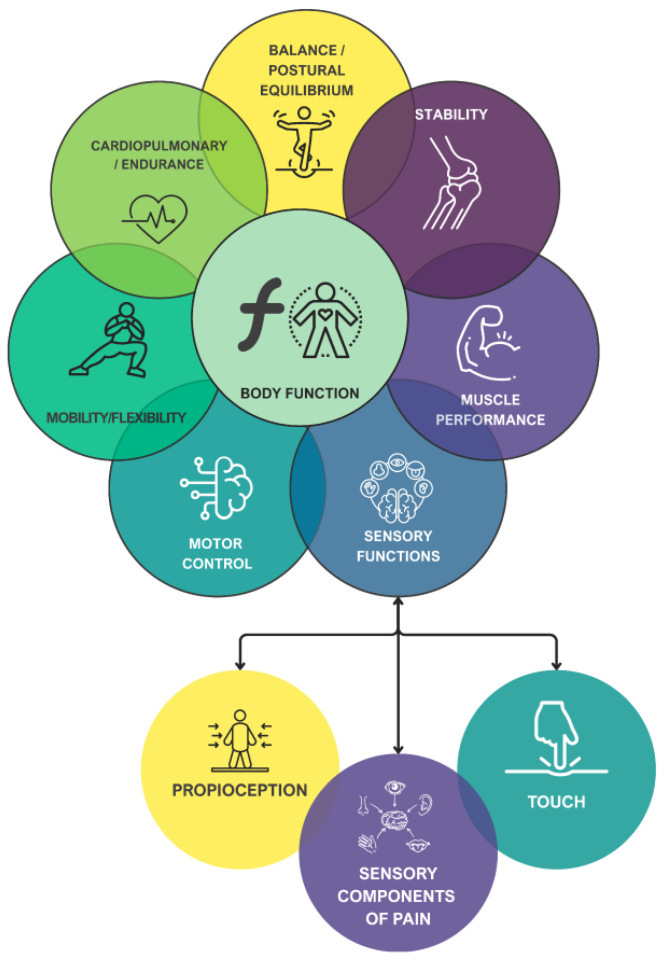
Multidimensional aspects of physical functions integrated into body functions section of ICF and adapted to patients with neurological conditions. The figure illustrates how neurophysiotherapy-relevant domains (adapted from Kisner and Colby) are nested within ‘neuromusculoskeletal and movement-related functions,’ creating a clinically actionable subdivision for physiotherapist assessment.

It is essential to emphasize that the classical model pertains to the “neuromusculoskeletal and movement-related functions.” Therefore, the remaining health professionals should assess the patient’s deficits in all other categories belonging to the body functions section (e.g., mental functions, voice and speech functions). Given the competences of physiotherapists, it may be of interest to carry out an adaptation of the classical model proposed by Kisner and Colby. This adaptation would include sensory functions within the model and a modification of motor control. The subsequent discussion will address this adaptation, resulting in a modified model of Kisner and Colby adapted to neurophysiotherapy, as illustrated in Figure 4.

In light of this approach, the physiotherapist must conduct a neurological examination by assessing the proposed domains. Table 1 summarizes these domains, their operational definitions, and recommended assessment tools. Each domain addresses a distinct aspect of physical function that can be impaired following neurological injury.

It is important to clarify the terminology employed in the context of motor control within the BRAIN framework. Kisner and Colby define neuromuscular control/coordination as ‘the interaction of the sensory and motor systems that enables synergists, agonists and antagonists, as well as stabilizers and neutralizers to anticipate or respond to proprioceptive and kinesthetic information and, subsequently, to work in the correct sequence to create coordinated movement’ [53,78]. This construct is identified in various terminologies throughout the extant literature. Within the domain of neurology, the term “motor control” is frequently employed to describe similar phenomena [79]. Broader definitions characterize motor control as ‘how the nervous system interacts with other body parts and the environment to produce purposeful, coordinated actions’ [80], emphasizing that movement results from a complex decision-making process involving sensorimotor and cognitive areas [81]. However, these definitions describe the integration of multiple systems to achieve functional, goal-directed tasks—a level of analysis that corresponds to the ‘activity’ domain of the ICF rather than to ‘body functions.’

This creates a conceptual gap: while the physical function domains proposed by Kisner and Colby adequately address musculoskeletal impairments, they do not explicitly capture a fundamental deficit observed in patients with upper motor neuron lesions—the loss of selective, fractionated movement. To address this gap, we propose an operationalized definition of motor control at the impairment level within the BRAIN framework.

In accordance with the conceptual framework developed by Krakauer et al. [26,82,83], the term “motor control at the body functions level” is defined as “the capacity for selective, independent joint movement.” That is to say, it signifies the ability to activate muscles in isolation and produce fractionated motion outside obligatory synergy patterns. This definition is intentionally limited and clinically anchored. It exemplifies a fundamental adverse indicator of upper motor neuron injury, specifically the impairment of fractionated movement due to corticospinal tract damage. This definition aligns with the assertions put forth by Krakauer et al. and other researchers [26,84,85,86].

Corticospinal tract lesions result in a characteristic loss of the ability to activate muscles independently. Instead, patients exhibit obligatory synergies, defined as fixed, stereotyped patterns of multi-joint co-activation [82,83]. These synergies are not compensatory strategies but indicate a fundamental motor constraint: the patient cannot voluntarily ‘break’ the synergy to produce isolated joint motion. Recent findings indicate that impaired dexterity and abnormal synergies are dissociable phenomena, meaning that one can be present without the other, underscoring that loss of fractionated control constitutes a distinct, measurable impairment [84].

Therefore, within the BRAIN framework, we distinguish between two levels: The first component of motor control is defined as the capacity for selective joint movement. This component can be assessed through validated tools such as the Fugl-Meyer Assessment or electromyographic analysis [26,76,77]. The second component is defined as the broader definition of motor control [34,53,78,79,80,81]. This component describes the integration of sensorimotor systems to produce goal-directed actions. This distinction provides clinicians with a clear, neurologically grounded intervention target at the impairment level, while maintaining coherence with established frameworks.

It is also important to note that the pain subdomain within body functions refers to altered nociception in the patient. However, a biopsychosocial assessment of pain should be conducted to evaluate the sensory-discriminative, emotional-affective, and cognitive-evaluative aspects of pain [87].

### 3.2. Activity and Participation Assessment

On the other hand, physiotherapy should specifically assess the domain d4 mobility of ICF. An evaluation of other domains necessitates a transdisciplinary, multidisciplinary, or interdisciplinary methodology, encompassing areas such as neuropsychology, occupational therapy, and speech therapy. This collaborative approach is essential for achieving a substantial impact on patients’ lives. People with neurological deficits represent a highly heterogeneous group, making the assessment of their functional capacity crucial for classifying their ability to perform various activities [88,89,90,91].

The Functional Ambulation Category (FAC), as shown in Table 2, enables clinicians to classify patients into six categories based on their ambulatory patterns.

**Figure 5:**
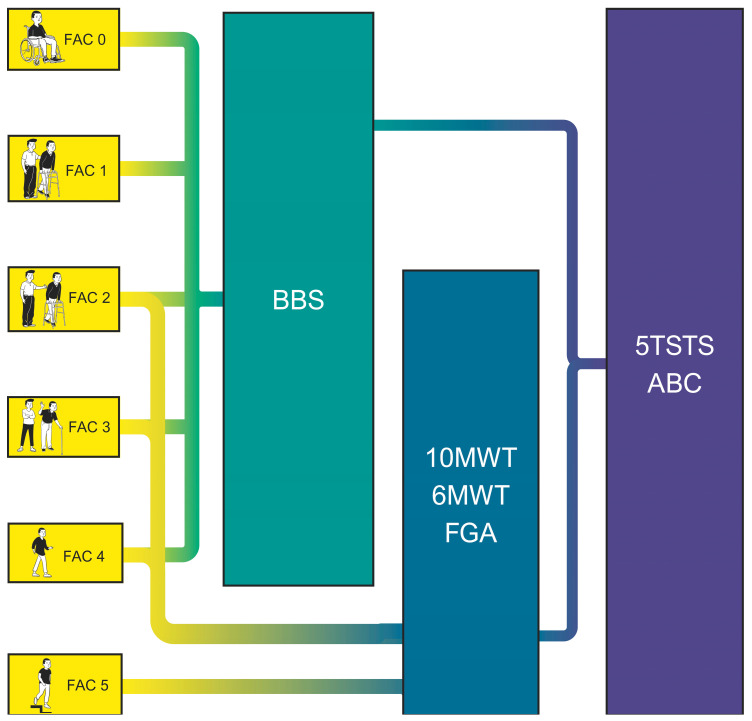
Activity assessments related to gait. FAC: Functional Ambulation Category; BBS: Berg Balance Scale; 10MWT: 10 Meter Walk Test; 6MWT: 6 Minute Walk Test; FGA: Functional Gait Assessment; 5TSTS: 5 Times Sit-to-Stand Test; ABC: Activities-Specific Balance Confidence Scale. Selection proceeds from left (FAC classification) to right (specific activity tests), ensuring assessments are matched to patient capability.

Thus, the selection of tests to assess the patient’s activity depends on the patient’s functional capacity and must be conducted in accordance with the recommendations made by the APTA and the Academy of Neurologic Physical Therapy [94] as reflected in Figure 5 and Supplementary Materials. In accordance with these guidelines, activities assessed by the physiotherapist are: balance during functional activities (Berg Balance Scale) [95,96,97,98]; walking balance (Functional Gait Assessment) [97,99]; balance confidence (Activities-specific Balance Confidence Scale) [100,101,102]; walking speed (10 Meter Walk Test) [103]; walking distance (6 Minutes Walking Test) [104,105,106]; and transfers (5 Times Sit-to-Stand) [107].

It is important to note that this classification system is exclusively designed for lower limb activities, so other assessments must be applied to evaluate upper limb activity. Instruments such as the Action Research Arm Test [108,109], the Box and Block Test [110,111] or the Nine-Hole Peg Test [112,113,114], are indicated for the assessment of upper limb activity in patients with neurologic deficits, provided that their functional capabilities allow for the administration of the test.

Lastly, participation is usually assessed through questionnaires such as the Stroke Impact Scale [115] or Canadian Occupational Performance Measure [116]. However, alternative scales can be used to evaluate the perceived participation of the patient in various activities of daily living, including the Motor Activity Log’s Amount of Use subscale, both in upper and lower limb versions [117].

### 3.3. Clinical Reasoning Workflow

The evaluation of this decision-making process provides the physical therapist with a clear picture of the patient’s deficits. However, neurologic patient management is complex, and it is nearly impossible to approach all these deficits simultaneously without diminishing treatment effectiveness. This underscores the rationale behind the framework’s alignment with the biopsychosocial paradigm and goal-oriented approach.

Within this process, both inductive and deductive reasoning interact dynamically. During the initial examination, clinicians rely primarily on inductive reasoning—pattern recognition based on clinical experience and prior encounters with similar presentations—to generate preliminary hypotheses about the patient’s impairments. Subsequently, clinicians employ deductive reasoning during the evaluation and diagnosis process. In this process, clinicians systematically test these hypotheses against clinical findings, established theoretical principles, and available evidence, to confirm or refute initial impressions. This iterative process continues throughout the rehabilitation process, as new information emerges or patient status changes, prompting clinicians to revisit their hypotheses and adjust their clinical decisions accordingly.

The decision-making process commences with a comprehensive evaluation of the patient’s limitations, activities, and participation in a broader context. This evaluation serves as the foundation for identifying the specific body functions that prevent the patient from achieving their goals. This requires adapting the classical APTA model to align with the specific requirements of physiotherapists treating patients with neurological impairments. A salient feature of the BRAIN framework is that goal setting is inherently a shared decision-making process. Clinicians and patients collaboratively negotiate therapeutic objectives, balancing patient preferences and life priorities with clinical feasibility and available evidence. When patient goals do not align with therapist recommendations—for example, when a patient’s expectations exceed realistic functional prognosis—the framework encourages transparent discussion of expected outcomes, potential risks, and alternative objectives. This approach ensures that final goals are both meaningful to the patient and clinically appropriate, adhering to SMART principles [46,47]. Consequently, the initial clinical reasoning process facilitates the identification of potential deficits that require attention at an early stage, even if they do not impede the immediate objective, with the aim of mitigating subsequent complications. However, if these deficits do not imply a long-term issue, the physiotherapist should focus on addressing the problems that prevent the patient from achieving their goals to improve participation and quality of life [46].

Furthermore, although the BRAIN framework focuses specifically on neurophysiotherapy-related domains (body functions, activity, and participation within the physiotherapist’s scope), successful neurological rehabilitation requires addressing contextual factors that influence outcomes. Psychological factors (e.g., depression, anxiety, self-efficacy, fear of movement), social factors (e.g., support systems, caregiver availability, environmental barriers), and behavioral factors (e.g., treatment adherence, health beliefs) are recognized as critical modulators of rehabilitation success. Within the ICF structure, these factors are classified as ‘personal factors’ and ‘environmental factors.’ The BRAIN framework acknowledges that systematic screening for these factors should be integrated into the examination phase, with appropriate referral to other team members (e.g., neuropsychology, social work, occupational therapy) when significant issues are identified. This multidisciplinary approach ensures comprehensive patient management.

**Figure 6:**
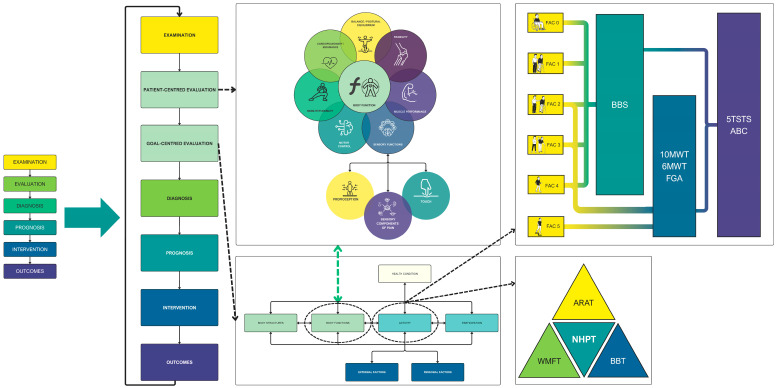
Biopsychosocial Reasoning Approach in Neurophysiotherapy (BRAIN) framework and workflow. This comprehensive flowchart integrates the ICF framework, APTA patient-management process, and adapted physical function domains. Clinicians begin with an examination across all domains (body functions, activity, participation), evaluate and diagnose impairments, set collaborative, patient-centered goals, select priority impairments that limit goal achievement, implement evidence-based interventions, and measure outcomes to iteratively refine the care plan.

The BRAIN framework provides a structured, multidimensional model for clinical reasoning in neurophysiotherapy. Grounded in the biopsychosocial paradigm, this approach integrates the ICF, a modified clinical decision-making process proposed by the APTA, and the interrelated physical function domains described by Kisner and Colby, adapted for neurophysiotherapy. Its goal-oriented structure has been shown to enhance patient-centered care, thereby supporting physiotherapists in making consistent, evidence-based decisions throughout the neurological rehabilitation process, as reflected in Figure 6. While other concepts have attempted to incorporate similar frameworks related to the biopsychosocial model [118], the lack of operationalization of these paradigms represents a gap in the literature that needs to be addressed to improve the effectiveness of physiotherapy treatments in clinical settings.

## 4. Novel Contributions of the BRAIN Framework

Although previous approaches have integrated the ICF into neurological rehabilitation [13,14,118], the BRAIN framework introduces several elements that aim to bridge the gap between theoretical models and clinical applicability. First, it provides an explicit, operationalized mapping between ICF ‘body functions’ and the neurophysiotherapy-specific domains of physical function (adapted from Kisner and Colby [53]), creating a direct translational bridge for clinicians. The framework under consideration proposes a clear distinction between ‘body functions’ and ‘body structures’ within the clinical reasoning process. This distinction allows physiotherapists to focus specifically on modifiable physiological impairments rather than on anatomical changes that may be less amenable to intervention.

Second, BRAIN proposes a unified language and a shared workflow for clinical reasoning in neurological physiotherapy. In contrast to prior NDT-based or experience-driven models, which have been criticized for lacking reproducibility and clear operational definitions [15,33], this framework provides a structured, teachable template that standardizes decision-making steps across the ICF, APTA patient-management process, and adapted physical function domains. This common language can facilitate interdisciplinary communication, clinical documentation, and educational training.

Third, the framework explicitly addresses the heterogeneity inherent to neurological populations by providing tiered, FAC-based assessment pathways for activity-level evaluation [92,93,94]. This ensures that test selection is matched to patient capability rather than applied uniformly.

Lastly, by embedding goal-oriented, patient-centered reasoning directly into the clinical workflow [52], BRAIN aims to ensure that clinicians systematically prioritize the impairments most limiting to patient-identified functional goals. This approach is aligned with contemporary rehabilitation principles but not yet operationalized within a unified neurophysiotherapy-specific framework. Collectively, these features position BRAIN as a structured, evidence-informed, and neurologically grounded framework that is both theoretically coherent and clinically actionable.

## 5. Limitations

Although the proposed framework offers a comprehensive approach to clinical reasoning in neurological physiotherapy, several limitations must be considered. First, the integration of the Kisner and Colby aspects of physical function and the ICF framework might require significant adaptation for use in diverse clinical settings, particularly in environments where multidisciplinary collaboration is less prevalent and physiotherapists have limited interaction with other healthcare professionals. However, it is imperative to incorporate this approach in the management of neurological patients.

Additionally, it is important to note that the proposed model is theoretical and conceptual in nature and currently lacks empirical validation. Although it is based on a comprehensive review of the scientific literature and established frameworks such as the ICF, the classical APTA model, and the interrelated aspects of physical function according to Kisner and Colby, it remains to be systematically validated through empirical evidence. Therefore, although it offers a coherent structure aligned with current trends in neurological rehabilitation, its practical applicability may vary depending on the clinical context and the degree of training of the professionals.

Likewise, it is recognized that studies are needed to validate and refine this model. Future research should prioritize the following steps to validate and refine this model: (1) application of expert consensus methods, such as the Delphi technique, with specialists in neurological physiotherapy to assess content validity and clinical relevance; (2) pilot implementation studies in diverse clinical settings to evaluate feasibility, acceptability, and potential barriers to adoption; and (3) comparative studies examining clinical outcomes (e.g., goal attainment, functional improvement, patient satisfaction) in settings using the BRAIN framework versus standard care. These strategies would allow assessment of the model’s practical utility and guide optimization of its structure based on real-world interdisciplinary requirements.

Further empirical studies and clinical trials are needed to validate the framework’s effectiveness and its potential impact on neurological rehabilitation outcomes across diverse patient populations and healthcare settings.

Conversely, all figures included in this manuscript have been designed using the viridis color palette, a perceptually uniform scale that optimizes visual accessibility. This palette offers several advantages over other traditional scales, as it maintains a progressive luminance and is suitable for people with color vision deficiencies, including color blindness [119]. Its use has been recommended in scientific visualization to enhance interpretive clarity, particularly in academic or clinical contexts where visual accuracy is crucial.

## 6. Conclusions

In conclusion, the present study proposes a comprehensive framework for clinical reasoning in neurological physiotherapy. This framework integrates the Kisner and Colby interrelated aspects of physical function within the classical APTA proposal and the ICF framework to create a more adaptable and patient-centered approach. The BRAIN framework is predicated on the biopsychosocial model and goal-oriented strategies. These elements empower physiotherapists to address the unique challenges of managing neurological patients, with a focus on enhancing patient participation and quality of life. The unified model under consideration encourages effective interdisciplinary collaboration and offers a clear pathway for improving rehabilitation outcomes, thereby addressing a gap in current clinical practice.

## Figures and Tables

**Table 1:** Body Functions Domains and Assessment Tools in the BRAIN Framework.

Domain	Subcomponent	Operational Definition	Assessment Tools	References
Muscle Performance	Strength	Capacity of muscle to produce tension and generate force	Handheld dynamometry, Manual Muscle Testing	[52,53,54,55,56]
	Power	Ability to produce force rapidly (strength × velocity)	Isokinetic dynamometry	[52,53,56,57]
Cardiopulmonary Endurance	—	Ability of cardiovascular and pulmonary systems to support sustained physical activity	Submaximal exercise tests obtain a value of VO2max	[58,59]
Mobility and Flexibility	Range of Motion	Available joint excursion (outcome measure)	Goniometry, electrogoniometry	[60,61]
	Structural limitation	Movement restriction due to joint capsule, muscle length, or connective tissue changes	End-feel assessment, muscle length tests,	[61,62]
	Neural hyperresistance	Velocity-dependent (spasticity) and velocity-independent (dystonia, rigidity) resistance to passive movement	MAS ^1^, MTS ^2^	[63,64]
Balance	—	Ability to distribute the body mass during functions	Posturography	[65,66]
Stability	Structural integrity	Anatomical and biomechanical integrity of joint structures	Clinical joint examination, stress tests	[67,68]
	Functional stability	Neuromuscular capacity to maintain joint stability during movement	Dynamic stability tests	[69]
Sensory Functions	Proprioception	Awareness of joint position and movement in space	Mirror Test, Proprioceptive subscale of FMA ^3^	[70]
	Touch	Perception of external stimuli	SW ^4^, TPD ^5^	[71,72]
	Pain	Nociceptive processing and pain perception	VAS ^6^, NRS ^7^, QST ^8^	[73,74,75]
Motor control *	—	Capacity for selective, independent joint movement; ability to activate muscles in isolation outside obligatory synergy patterns	FMA ^3^, kinematic or Electromyographic analysis	[26,76,77]

* Operationally defined at the impairment level (ICF body functions). This definition should not be confused with broader, task-level conceptions of motor control that describe the interaction among the individual, task, and environment to produce coordinated actions, corresponding to the activity level of the ICF. See the text for the full rationale; ^1^: Modified Ashworth Scale; ^2^: Modified Tardieu Scale; ^3^: Fugl-Meyer Assessment; ^4^: Semmes-Weinstein Monofilaments; ^5^: Two Point Discrimination Test; ^6^: Visual Analogue Scale; ^7^: Numeric Rating Scale; ^8^: Quantitative Sensory Testing.

**Table 2:** Functional Ambulation Category (FAC) descriptions [92,93].

FAC Level	Ambulation Capacity	Description
0	Nonfunctional ambulator	The patient is not able to walk at all or needs the help of two therapists
1	Ambulator dependent on physical assistance (level II)	The patient requires continuous manual contact to support body weight as well as to maintain balance or to assist coordination
2	Ambulator dependent on physical assistance (level I)	The patient requires intermittent or continuous light touch to assist balance or coordination
3	Ambulator, dependent on supervision	The patient can ambulate on a level surface without manual contact of another person, but requires standby guarding of one person either for safety or for verbal cueing
4	Ambulator, independent	Level surface only: the patient can ambulate independently but requires supervision to negotiate (e.g., stairs, inclines, nonlevel surfaces)
5	Ambulator, independent	The patient can walk everywhere independently, including stairs

## Data Availability

No new data were created or analyzed in this study.

## Supplementary Materials

The following supporting information can be downloaded at: https://www.mdpi.com/article/10.3390/brainsci16020235/s1, Table S1: Activity and Participation Assessment Tools.

## Abbreviations

The following abbreviations are used in this manuscript:

10MWT	10 Meter Walking Test
5TST	5 Times Sit to Stand Test
6MWT	6 Minutes Walking Test
APTA	American Physical Therapy Association
ARAT	Action Research Arm Test
ABC	Activities-specific Balance Confidence Scale
BBS	Berg Balance Scale
BBT	Box and Block Test
BRAIN	Biopsychosocial Reasoning Approach In Neurophysiotherapy
ESO	European Stroke Organization
FAC	Functional Ambulation Category
FGA	Functional Gait Assessment
FMA	Fugl-Meyer Assessment
GAS	Goal Attainment Scale
ICF	International Classification of Functioning, Disability and Health
MAL	Motor Activity Log
MAS	Modified Ashworth Scale
MTS	Modified Tardieu Scale
NDT	Neurodevelopmental Therapies
NHPT	Nine-Hole Peg Test
NRS	Numeric Rating Scale
QST	Quantitative Sensory Testing
SMART	Specific, Measurable, Achievable/Attainable, Realistic/Relevant, Timed
SW	Semmes-Weinstein
TPD	Two-Point Discrimination Test
VAS	Visual Analogue Scale
VO_2_max	Maximum Oxygen Consumption
WHO	World Health Organisation
WMFT	Wolf Motor Function Test
