# Supplementary material for: Advancing Neurological Rehabilitation: The BRAIN Framework for Clinical Reasoning in Neurophysiotherapy
Source: Brain Sci. 2026 Feb 18;16(2):235. doi: 10.3390/brainsci16020235 (PMC12938968; doi:10.3390/brainsci16020235)
Supplement: Supplementary file 1 [file brainsci-16-00235-s001.zip › brainsci-4133266-supplementary.pdf]

Supplementary Material

Table S1. Activity and Participation Assessment Tools

ICF Level	Domain	Functional Category	Assessment Tools
Activity	*Mobility (d4)*	Non-Ambulatory (FAC[1] 0-1)	BBS[2], 5TST`[3], ABC[4]
Activity	*Mobility (d4)*	Assisted ambulation (FAC 2-4)	BBS, 5TST, ABC, 10MWT[5], 6MWT[6], FGA[7]
Activity	*Mobility (d4)*	Independent ambulation (FAC 5)	5TST, ABC, 10MWT, 6MWT, FGA
Activity	*Upper limb*	Gross motor function	ARAT[8], BBT[9]
Activity	*Upper limb*	Fine motor function/dexterity	NHPT[10]
Participation	*Social involement*	Self-perceived participation	SIS[11], COPM[12], MAL[13]

1: Functional Ambulation Category; 2: Berg Balance Scale; 3: 5 Times Sit-to-Stand; 4: Activities-specific Balance Confidence Scale; 5: 10 Meter Walk Test; 6: 6 Minutes Walking Test; 7: Functional Gait Assessment; 8: Action Research Arm Test; 9: Box and Block Test; 10: Nine Hole Peg Test; 11: Stroke Impact Scale; 12: Canadian Occupational Performance Measure; 13: Motor Activity Log
